# Using FaceReader to explore the potential for harnessing emotional reactions to motivate hand hygiene

**DOI:** 10.1177/17571774211060394

**Published:** 2022-02-22

**Authors:** Sophie Rutter, Marc Bonne, Catherine Stones, Colin Macduff

**Affiliations:** 1 Information School, 152609 The University of Sheffield, Sheffield, UK; 2 4468 University of Leeds, Leeds, UK; 3 3517 The Glasgow School of Art, Glasgow, UK

**Keywords:** handwashing, hand hygiene, messaging, emotion, disgust

## Abstract

**Background:**

Handwashing is a key strategy for reducing the spread of infection but hand hygiene practises are often poor. Pre-testing messages prior to a campaign is expensive and time consuming.

**Objective:**

This study investigates (1) emotional reactions to handwashing messages based on four different theoretical constructs (Knowledge of Risk, Comfort, Disgust and Social Norms), (2) how images may influence emotional reactions and (3) the influence of emotion, images and theoretical construct on handwashing motivation.

**Methods:**

A novel methodology was employed whereby FaceReader, software that automatically analyses emotions, was used to identify reactions to handwashing messages. Thirty-one participants from The University of Sheffield were recruited for this laboratory study.

**Results:**

Most participants did not react strongly to any message and emotional reactions were similar for messages from different theoretical constructs. Adding images to text messages intensified some emotional reactions, particularly Happy and Disgusted for the two messages from the Disgust theoretical perspective. Moreover, participants thought that messages that used images were 1.8 times more likely to encourage handwashing. Knowledge of Risk messages (most encouraging) were 2.9 times more likely to be selected as encouraging handwashing than Comfort messages (least encouraging). An increase in the Disgusted emotion was also associated with an increase in encouragement.

**Discussion:**

This study suggests that handwashing messages should be designed to exploit emotional reactions but more research is needed to understand how to design messages for these reactions. Whether disgust is as important post Covid-19 requires future investigation. FaceReader can be usefully and inexpensively employed to pre-test handwashing messages.

## Background

Handwashing with soap reduces the risk of infection-related illness (World Health Organization, 2020). Historically hand hygiene is poor with as little as 19% of the global population thought to wash their hands after using toilet facilities (Freeman et al., 2014). In response to the Covid-19 pandemic there has been an increased emphasis on handwashing in both national and international campaigns (e.g. Department of Health and Social Care, 2020), but it is not known which campaigns have been effective. Randomised controlled trials (RCT) are considered the gold standard for evaluating the effectiveness of handwashing campaigns. However, RCTs are expensive to run. Furthermore, it is impracticable to test multiple variations of a campaign (Judah et al., 2009). In this paper, we explore a novel approach to evaluating handwashing messages prior to their implementation in campaigns.

Emotional reactions to 32 handwashing messages from four different theoretical perspectives were measured using face reading software (that automatically recognises and numerically analyses facial expressions). Whether participants’ emotional reactions to messages impacted on their intention to wash hands was also investigated.

This study is informed by the work of Judah et al. (2009) where the effectiveness of two handwashing messages for seven different theoretical constructs were tested on the general public in motorway service station washrooms. The Judah et al. (2009) study found that for most constructs, there was a small but significant increase in soap consumption. Knowledge Activation, Knowledge of Risk and Positive Control messages were particularly effective for women. Disgust and Norm messages were particularly effective for men. Judah et al. (2009) suggest women may have reacted differently because the mention of germs in the knowledge-based messages may have prompted a disgusted response. Therefore, it could be that messages are not eliciting the response that the message designer intended to provoke, and that people’s reactions vary depending upon their past experiences and other factors. This is further evident as the effectiveness of the two messages within a construct varied, particularly for Comfort and Social Norms. In a study of healthcare workers, Taylor (2017) found that the effectiveness of different message strategies varied depending on their execution. Images are likely to provoke more emotional reactions compared with text which provokes more rational, logical and linear thought (Joffe, 2008) but their role in communicating hand hygiene is not well understood.

Accordingly, there is a need to further explore and clarify the relationships between reactions and the content and format of messages. This study addresses this need by using novel methods to answer the following research questions:

• RQ1: Do handwashing messages based on different theoretical constructs produce different emotional reactions?
• RQ2: Does adding an image to a text message change the emotional reaction?
• RQ3: Is there a relationship between an emotional reaction and participants’ intention to wash their hands?

## Methods

### Study design overview

**Figure 1.**
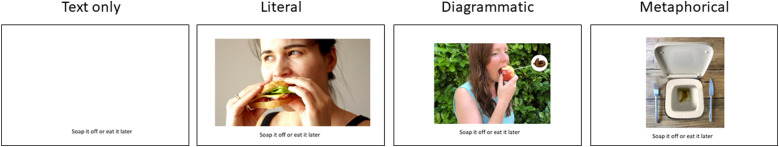
Example message formats for one of the two Digust messages.

Messages from four of seven theoretical constructs used in the Judah et al. (2009) study were selected (Knowledge of Risk, Comfort, Disgust and Social Norms). The others were omitted because in some instances it was difficult to find images to illustrate the connotation of the message (e.g. for the knowledge activation message “Wsah your hands wiht soap” the recipient must descramble the words and there is no obvious image that could support this knowledge activation), and because using the full seven seemed very likely to induce participant fatigue. For each of the four theoretical constructs the two messages used in the original study were reproduced verbatim. Additionally, each message was illustrated using three types of images: literal, diagrammatic and metaphorical (Figure 1). The rationale for the three types of image was that each image type, when anchored by the text, operates at a different level of meaning and requires different levels of cognitive processing. The literal image illustrates broadly the subject of the message and only requires basic recognition. The diagrammatic message requires recognising the connection between literal elements and illustrates the contents of the message specifically. The metaphorical message requires making more novel and dramatic connections between the visual elements and the viewer’s experiences in the world. It illustrates the contents of the message specifically but attempts to add further real-world associations.

A within-subjects design was employed with the message order rotated for each participant. Thus, each participant viewed every message but in a different order so the results can be attributed to the message viewed and not the order of messages.

### Recruitment and participant sample

An email was sent via the The University of Sheffield volunteer email list inviting potential participants to take part in a study evaluating handwashing messages and images. Participants received a £10 voucher as an honorarium for their time spent. 31 participants were recruited. Participants were mainly young, well-educated and either studying or working at The University of Sheffield. Seven participants were under 25 years of age, 18 were 25–34, 4 were 35–44 and 2 were 45+. 20 participants were female and 11 male. Participants came from diverse cultural backgrounds, with only a third of students having English as their first language: 7 students speaking Arabic, 5 Mandarin, 3 Spanish, 3 Italian, 1 Russian and 1 Serbian as their first language. Therefore, although participants in this study were recruited from one setting, there is considerable demographic diversity.

### Research instruments and data collection procedure

The study took place in the The University of Sheffield research lab in June 2019, prior to the Covid-19 pandemic. After informed consent was received, participants were asked to complete a brief demographics questionnaire (age, gender, home country and first language). Participants were then shown the randomised message sample using PowerPoint. Participants viewed each message for 8 seconds. Emotional reactions to the messages were recorded and measured with Noldus FaceReader. FaceReader automatically analyses emotions using the Facial Action Coding System developed by Ekman and Friesen (1978). FaceReader has been validated as 88% accurate but marginally better at recognising female emotions (89%) than male (86%) (Lewinski et al., 2014).

Happy, Sad, Angry, Surprised, Scared and Disgusted are the six basic emotions (i.e. the building blocks of all emotional reactions) and are considered universal (Ekman and Cordaro, 2011). FaceReader records these six emotions as well as Neutral, Valence and Arousal. The intensity of an emotional reaction is recorded on a scale of 0–1. An intensity of 0.2 is considered slightly visible and 0.5 clearly visible (Kuilenburg et al., 2005). Valence can vary between +1 and −1 and is calculated as the intensity of Happy minus the intensity of the negative emotion (i.e. Angry, Sad, Disgusted and Scared) with the highest intensity. Surprised can be either positive or negative, so is not included in valence measurements.

To identify the potential effectiveness of messages participants were shown a summary sheet of all messages and were asked to ‘select which of these messages would/would not encourage you to wash your hands’. Participants were told they did not need to give a response for each message if they were unsure. Asking participants’ opinions has been used in other similar studies (e.g. Taylor, 2017) to pre-test multiple measures.

### Data analysis

Mean valence was used to identify each participant’s overall emotional reaction for each message type. Emotional reactions are fleeting (Ekman, 1992) so maximum intensity was used to identify the strength of specific emotions for the different messages.

Data were not normally distributed so non-parametric statistical tests were used to test differences between theoretical constructs, message format, gender and participant opinion.

(1) Wilcoxon Signed Rank tests were used to test differences in Valence and intensity of the six basic emotions for the two messages within a theoretical construct (RQ1), as well as differences in Valence and the six basic emotions between a text message and other formats within a theoretical constructs (RQ2)
(2) Friedman tests were used to test differences in Valence and intensity of basic emotions across the four theoretical constructs (RQ1).
(3) Mann Whitney U tests were used to test for gender differences (RQ1)
(4) Binary Logistic Regression was used to identify factors influencing participants’ opinions (RQ3)

It should be noted that as there are 32 conditions and multiple tests, it is possible that there will be type I errors. As the study is exploratory, and as with the Judah et al. (2009) study, the *p* value has not been adjusted as this could then lead to type II errors. Instead, a more descriptive approach is taken with the *p* values and significance levels interpreted with some caution. Furthermore, alternative interpretations of the results are offered (Brandt, 2007; Perneger, 1998).

### Limitations

Reactions to messages might be different in locations where handwashing takes place. Judah et al. (2009) found that messages were more effective for men when washrooms were busier probably because people are more likely to wash their hands when others are present. An advantage of conducting the study in a laboratory is that the testing of messages is highly controlled and so the same conditions apply to all messages.

The sample size is normal for a laboratory study but too small to test for how age and nationality might account for different emotional reactions. The participants were all attendees of Higher Education and therefore likely have a higher than average cognitive ability for processing information. Further tests would be required on a sample that more typically represents the population of the UK.

Each of the eight messages was illustrated with three image variations. Other characteristics of images could also be usefully tested. For example, in a study of hand sanitiser usage in clinical environments, King et al. (2016) found that placing an image of a male eye above a hand sanitiser increased usage but an image of a female eye did not. While it was not practical to test different representations of images in this study that already had a large number of variables, we were careful to vary image representations across the study.

### Ethics

All subjects gave their informed consent before they participated in the study. All data was anonymised to ensure confidentiality. The study was approved (reference number 026624) by the Ethics Committee at The University of Sheffield on 5 June 2019.

## Results

Two FaceReader recordings failed. Once because the FaceReader application crashed mid recording and once because the participant partially obscured their face with their hand. Therefore, the data from 29 participants were used to answer RQ1 and RQ2. A further two participants spoilt their summary sheets of all messages and these were removed from analysis for RQ3.

Results are reported in Supplemental Material.

### Do handwashing messages based on different theoretical constructs produce different emotional reactions? (RQ1)

The two text messages from each of the four theoretical constructs are analysed for (1) similarities in emotional reaction between messages from the same construct, and (2) differences in emotional reaction between messages from different constructs.

For all four theoretical constructs and message variations, Valence is slightly negative. Wilcoxon Signed Rank tests confirmed that there were no significant differences in Valence for the two messages within a theoretical construct indicating that emotional reactions were similar. A Friedman test confirmed that there were no significant differences in Valence across the four theoretical constructs, also indicating that emotional reactions were similar regardless of theoretical construct. Mann Whitney U tests found no significant differences based on gender.

Maximum intensity was low for all six basic emotions regardless of theoretical construct, suggesting that most participants did not react strongly to any message. Wilcoxon Signed Rank tests confirmed that there were no significant differences in the intensity of emotions for the two text only messages from within each theoretical construct, except for Happy between the two Disgust theoretical construct messages. A type 1 error could account for this particularly given the number of tests. A Friedman test confirmed that there were no significant differences in emotional reactions across different theoretical constructs. Mann Whitney U tests found that females had significantly higher intensity scores for some emotions and messages.

The results of these tests suggest that handwashing messages based on different theoretical constructs are not producing different emotional reactions (RQ1).

### Does adding an image to a text message change the emotional reaction? (RQ2)

Emotional reactions are compared for the different formats of each of the message.

Valence for all messages formats is slightly negative. Wilcoxon Signed Rank tests confirmed that there were no significant differences in Valence between a text message and other formats within a theoretical construct. Gender differences were not tested for significance as previous studies have not tested gender differences for message formats, and so there is no theoretical basis.

Wilcoxon Signed Rank tests indicate that adding an image to a text message can significantly alter the intensity of some of the basic emotions for some of the message variations particularly those from the Disgust theoretical construct. As so many tests were performed some caution needs to be taken when interpreting this result given the possibility of a type 1 error.

The results of these tests suggest that adding an image to a text only message intensifies some of the emotional reactions for some message variations (RQ2).

### Is there a relationship between an emotional reaction and participants’ intention to wash their hands? (RQ3)

The messages are analysed to identify whether there is a relationship between emotional reaction and participants’ opinion as to whether the messages would encourage handwashing.

Across the dataset, only 34% messages were selected as encouraging. Using binary logistic regression, the relationship between construct, message format, gender and emotion with what participants thought would likely encourage handwashing were tested. The overall model was statistically significant when compared to the null model, (χ2 (12) = 64.018, *p* < .001), explained 10% of the variation of survival (Nagelkerke R2) and correctly predicted 67% of cases. Construct (*p* < .001), Message format (*p* = .001) and a Disgusted emotional reaction (*p* < .001) were significant but Gender (.634), Valence (.455), Happy (.881), Sad (.840), Angry (.336), Surprised (.533) and Scared (.634) were not. Participants thought that messages that used images were 1.8 times more likely to encourage handwashing. As well, Knowledge of Risk messages were 2.865 times more likely to encourage handwashing than Comfort messages, and Disgust messages were 1.549 times more likely to encourage handwashing than Comfort. An increase in the Disgusted emotion was associated with an increase in encouragement. However, very little variation is explained with these variables; other factors are also affecting participants’ opinions.

The results of these tests suggest that there is a relationship between the Disgusted emotional reaction and what participants think will encourage them to wash their hands. Furthermore, theoretical construct and message format are also thought to influence handwashing intention.

## Discussion

The implications of the findings are now discussed. As this study was conducted prior to the Covid-19 pandemic, we consider whether emotional reactions to hand hygiene messaging and key drivers could change in a post-pandemic setting.

FaceReader was deployed to identify emotional reactions to 32 message variations across four theoretical constructs. Messages designed for Knowledge of Risk were most likely to be viewed as encouraging handwashing and Comfort messages were least likely. This finding is consistent with other studies that have compared the effectiveness of different message types (Judah et al., 2009; Taylor, 2017). Hand hygiene has increased during the Covid-19 pandemic (Office for National Statisics ONS, 2021) with Knowledge of Risk the likely initial motivator. That the results are similar to the other studies and recent campaigns is promising as it indicates that the methods used in this study are credible, and that lab-based studies are a good precursor to more expensive and time-consuming evaluations such as RCTs. FaceReader could be used to pre-test a large number of messages to get an initial indication of the most promising text/image combinations.

Emotional reactions were generally consistent across the different theoretical constructs (RQ1). This finding is unexpected as it was anticipated that different theoretical constructs would provoke different emotional reactions. For example, messages from a Comfort theoretical perspective might generate a stronger happy emotion and messages from a Disgust theoretical perspective a stronger disgusted emotion. It could be that the study inhibited emotional reactions because the situation was ‘not real’. However, it is also possible that the messages are not provoking the intended responses (Taylor, 2017). In future studies it could be beneficial to test message variations for emotional responses prior to roll out.

Previous research has found that gender influenced emotional reactions (Cameron et al., 2018) and handwashing (Judah et al., 2009). While there is some evidence for gender differences in our study, it is likely that other factors were more influential. Furthermore, gender did not influence participants’ selection of messages that encourage handwashing.

Adding images to text messages intensified some emotional reactions, particularly Happy and Disgusted for the two messages from the Disgust theoretical perspective (RQ2). That only a third of our participants had English as a second language could be a contributing factor here. Combining images with text has been used to effectively communicate hand hygiene to primary school children (age 4–11) (Rutter et al., 2020). Further work could usefully be done to identify what images people find disgusting/pleasing in a post-Covid-19 setting and how this might vary by cultural context. Some images used during later Covid-19 campaigns relied heavily on emotional triggers for their impact (Owen, 2021). For instance, a campaign (by Freuds for PHE, UK) launched in January 2021 featured dark close-up shots of Covid patients anchored by emotive text such as ‘Look her in the eyes and tell her you never bend the rules’ (Owen, 2021). A public information campaign from the UK’s Department of Health and Social Care (DHSC, 2020) featured solarised/infrared images of hands touching green germ-filled handles in an attempt to use disgust as a trigger. Though it is unclear how effective these approaches have been, such emotive images would clearly benefit from prior testing using techniques described above.

Furthermore, because messages selected as encouraging had higher Disgusted reactions it may be beneficial to design messages that produce a Disgusted response (RQ3). This finding is consistent with three other studies that have used disgust images to promote handwashing with adults (Botta et al., 2008; Judah et al., 2009; Porzig-Drummond et al., 2009). That disgust is a universal motivator could also help explain why this particular emotion was so effective (Curtis et al., 2009). It should be noted, this study was conducted prior to the Covid-19 pandemic, and whether disgust is still an important motivator requires further investigation, particularly as Knowledge of Risk has likely driven the initial increase in public hand hygiene but this initial increase has not been fully sustained. Despite a plethora of messages and campaigns about hand hygiene, public compliance with hand hygiene is already decreasing (Office for National Statisics ONS, 2021) and there is an urgent need to identify effective messaging that works in the long term recommendations.

The results of this study also suggest that more could be done to exploit emotional reactions in handwashing campaigns. Further research is urgently needed to understand the different reactions to handwashing messages and images, and what motivates the public to wash their hands, particularly in a post Covid-19 setting.

The following recommendations are made

• Prior to an intervention messages should be pre-tested to check that they are provoking the intended emotional response
• FaceReader can be used to pre-test a large number of messages to get an initial indication of emotional response, and the most promising text/image combination
• Images should be combined with text to communicate hand hygiene.
• Messages that produced a Disgusted emotional response were perceived as effective drivers in this study. However, whether disgust is still as important as a motivator post Covid-19 requires future investigation.

## Supplemental Material

sj-pdf-1-bji-10.1177_17571774211060394 – Supplemental Material for Using FaceReader to explore the potential for harnessing emotional reactions to motivate hand hygiene Click here for additional data file. Supplemental Material, sj-pdf-1-bji-10.1177_17571774211060394 for Using FaceReader to explore the potential for harnessing emotional reactions to motivate hand hygiene by Sophie Rutter, Marc Bonne, Catherine Stones and Colin Macduff in Journal of Infection Prevention
