# Supplementary material for: Using FaceReader to explore the potential for harnessing emotional reactions to motivate hand hygiene
Source: J Infect Prev. 2022 Feb 22;23(3):87–92. doi: 10.1177/17571774211060394 (PMC9052853; doi:10.1177/17571774211060394)
Supplement: sj-pdf-1-bji-10.1177_17571774211060394 – Supplemental Material for Using FaceReader to explore the potential for harnessing emotional reactions to motivate hand hygiene [file sj-pdf-1-bji-10.1177_17571774211060394.pdf]

Supplementary Material

Table 1: Mean Valence and Maximum Intensity of Specific Emotions

- Differences between text and other message formats. MF**significant at 5% level; MF ***significant at 1% level
- Differences between theoretical constructs. TC** significant at 5% level.
- Differences between gender. G** significant at 5% level.

	Text		Literal		Diagrammatic		Metaphorical		Text		Literal		Diagrammatic		Metaphorical	
	Median	IQR (25, 75)[1]	Median	IQR (25, 75)	Median	IQR (25, 75)	Median	IQR (25, 75)	Median	IQR (25, 75)	Median	IQR (25, 75)	Median	IQR (25, 75)	Median	IQR (25, 75)
	**Comfort M1**								**Comfort M2**							
Happy	0.012 G**	0.002, 0.047	0.027	0.007, 0.228	0.017	0.001, 0.079	0.012	0.003, 0.529	0.018	0.002, 0.146	0.013	0.001, 0.211	0.028	0.002, 0.312	0.017	0.004, 0.323
Sad	0.048	0.017, 0.285	0.018	0.007, 0.193	0.042	0.006, 0.279	0.061	0.013, 0.215	0.034	0.011, 0.177	0.041	0.014, 0.173	0.033	0.013, 0.218	0.026	0.009, 0.158
Angry	0.058	0.02, 0.14	0.062	0.008, 0.182	0.051	0.016, 0.211	0.065 MF**	0.033, 0.212	0.052	0.007, 0.173	0.049	0.012, 0.189	0.070	0.022, 0.124	0.052	0.019, 0.199
Surprised	0.019	0.008, 0.088	0.050	0.013, 0.175	0.056 MF**	0.014, 0.133	0.015	0.005, 0.099	0.016	0.008, 0.059	0.030	0.005, 0.156	0.021	0.008, 0.159	0.021	0.007, 0.099
Scared	0.003 G**	0.001, 0.019	0.010	0.002, 0.031	0.005	0.002, 0.017	0.006	0.001, 0.026	0.007 G**	0.001, 0.018	0.006	0.001, 0.022	0.017 MF**	0.001, 0.037	0.011	0.002, 0.031
Disgusted	0.032	0.006, 0.253	0.029	0.006, 0.131	0.025	0.007, 0.118	0.024	0.006, 0.167	0.040	0.008, 0.245	0.044	0.016, 0.201	0.022	0.007, 0.117	0.028	0.008, 0.222
Valence	-0.085	-0.231, -0.027	-0.115	-0.223, -0.018	-0.102	-0.164, -0.01	-0.068	-0.236, 0.04	-0.095	-0.222, -0.011	-0.082	-0.177, -0.001	-0.111	-0.219, 0.006	-0.096	-0.207, 0.053
	Knowledge M1								Knowledge M2							
Happy	0.023	0.003, 0.293	0.015	0.002, 0.208	0.011	0.002, 0.139	0.024	0.004, 0.393	0.023 G**	0.005, 0.115	0.027	0.001, 0.214	0.024	0.003, 0.166	0.009	0.002, 0.07
Sad	0.077	0.028, 0.221	0.061	0.013, 0.197	0.078	0.011, 0.161	0.034	0.012, 0.174	0.041	0.011, 0.187	0.025	0.007, 0.19	0.058	0.013, 0.222	0.024	0.008, 0.256
Angry	0.072	0.023, 0.141	0.080	0.018, 0.132	0.057	0.02, 0.181	0.082	0.018, 0.162	0.064	0.024, 0.204	0.076	0.014, 0.194	0.080	0.008, 0.146	0.056	0.008, 0.204
Surprised	0.022	0.003, 0.096	0.026	0.008, 0.125	0.019	0.01, 0.112	0.026	0.006, 0.061	0.034	0.013, 0.09	0.032	0.006, 0.16	0.014	0.006, 0.071	0.027	0.004, 0.099
Scared	0.004 G**	0.001, 0.035	0.005	0.001, 0.018	0.008	0.002, 0.028	0.006	0.001, 0.024	0.015	0.002, 0.032	0.009	0.002, 0.023	0.008	0., 0.021	0.004 MF**	0.001, 0.022
Disgusted	0.045	0.018, 0.206	0.028 MF**	0.009, 0.128	0.031	0.009, 0.198	0.025	0.006, 0.237	0.034	0.015, 0.19	0.027	0.006, 0.156	0.042	0.006, 0.082	0.029	0.007, 0.071
Valence	-0.092	-0.274, -0.017	-0.088	-0.247, 0.012	-0.106	-0.385, 0.054	-0.146	-0.244, -0.025	-0.087	-0.251, -0.009	-0.072	-0.237, 0.054	-0.099	-0.167, -0.024	-0.107	-0.168, 0.14
	Norms M1								Norms M2							
Happy	0.020 G**	0.001, 0.091	0.014	0.003, 0.153	0.008	0.001, 0.136	0.012	0.001, 0.316	0.024	0.004, 0.475	0.012	0.001, 0.057	0.012	0.001, 0.149	0.023	0.003, 0.194
Sad	0.054	0.015, 0.231	0.055	0.01, 0.173	0.031	0.011, 0.147	0.021 MF**	0.008, 0.166	0.132	0.038, 0.308	0.035	0.015, 0.163	0.058	0.007, 0.202	0.106	0.012, 0.238
Angry	0.075	0.031, 0.147	0.038 MF**	0.024, 0.11	0.090	0.012, 0.176	0.084	0.015, 0.161	0.061	0.021, 0.252	0.072	0.016, 0.204	0.079	0.02, 0.218	0.098	0.014, 0.264
Surprised	0.021 G**	0.009, 0.157	0.011	0.005, 0.078	0.027	0.008, 0.094	0.022	0.009, 0.135	0.024	0.006, 0.172	0.024	0.007, 0.277	0.017	0.004, 0.037	0.047	0.007, 0.149
Scared	0.012 G**	0.002, 0.017	0.007	0.001, 0.015	0.004	0.001, 0.02	0.004	0.001, 0.013	0.006	0.001, 0.055	0.005	0.001, 0.016	0.003 MF**	0.001, 0.013	0.003	0.001, 0.026
Disgusted	0.023	0.01, 0.193	0.042	0.011, 0.09	0.013 MF**	0.006, 0.17	0.032	0.01, 0.18	0.086	0.017, 0.407	0.018 MF***	0.005, 0.096	0.036 MF**	0.004, 0.183	0.034	0.01, 0.17
Valence	-0.112	-0.239, -0.018	-0.095	-0.285, -0.013	-0.068	-0.235, -0.028	-0.093	-0.296, 0.011	-0.088	-0.175, 0.008	-0.137	-0.294, -0.018	-0.136	-0.273, -0.018	-0.122	-0.272, -0.004
	Disgust M1								Disgust M2							
Happy	0.014 TC**	0.002, 0.207	0.011	0.003, 0.084	0.151 MF***	0.018, 0.761	0.174 MF***	0.018, 0.717	0.010 TC** G**	0.001, 0.042	0.012 MF**	0.002, 0.51	0.020 MF**	0.004, 0.587	0.044	0.002, 0.243 MF***
Sad	0.039	0.024, 0.312	0.037 MF**	0.007, 0.183	0.073	0.013, 0.176	0.082	0.01, 0.234	0.051	0.014, 0.24	0.046	0.018, 0.196	0.058	0.016, 0.193	0.038	0.016, 0.206
Angry	0.048	0.016, 0.124	0.052	0.023, 0.168	0.056 MF**	0.019, 0.332	0.037	0.019, 0.137	0.062	0.025, 0.11	0.069	0.014, 0.155	0.053	0.022, 0.203	0.043	0.012, 0.159
Surprised	0.014	0.004, 0.081	0.024	0.007, 0.09	0.016	0.002, 0.101	0.014	0.004, 0.088	0.023	0.009, 0.052	0.027 MF**	0.007, 0.109	0.035 MF**	0.013, 0.092	0.028 MF**	0.005, 0.208
Scared	0.007 G**	0.001, 0.022	0.004	0.002, 0.017	0.003	0.001, 0.027	0.006	0.001, 0.028	0.003	0.001, 0.014	0.008	0.001, 0.02	0.005	0.002, 0.027	0.005	0.001, 0.016
Disgusted	0.029	0.008, 0.245	0.023	0.01, 0.125	0.076 MF**	0.029, 0.274	0.073 MF**	0.024, 0.368	0.029	0.006, 0.253	0.029	0.006, 0.22	0.027	0.009, 0.254	0.027	0.007, 0.287
Valence	-0.073	-0.231, 0.02	-0.109	-0.275, -0.032	-0.029	-0.2, 0.095	-0.018	-0.233, 0.165	-0.117	-0.278, -0.035	-0.047	-0.234, 0.119	-0.095	-0.184, 0.078	-0.088	-0.297, -0.024

[1] Interquartile range (25th ,75th percentile)
